# Impact of COVID-19 on Water Quality Index of river Yamuna in Himalayan and upper segment: analysis of monsoon and post-monsoon season

**DOI:** 10.1007/s13201-022-01625-3

**Published:** 2022-04-15

**Authors:** Shiwani Sharma, AnuShri Gupta

**Affiliations:** Department of Chemistry, School of Sciences, Abhilashi University, Chailchowk, Mandi, Himachal Pradesh 175028 India

**Keywords:** Physico-chemical parameters, Water quality index, River Yamuna, Water pollution, Himalayan and upper segment, COVID-19

## Abstract

Rivers are the lifeline of every living being, be it humans or animals. Clean water is essential for everyone. However, increased urbanization and rapid industrialization have led to rising pollution level in rivers. COVID-19 on the contrary has changed the entire ecosystem. Limited industrial activities, reduced people movement during COVID times has led to improvement in environment, be it atmosphere or hydrosphere. Present work aims to study the impact of COVID-19 on water quality index of river Yamuna as it traverses from Himalayan segment to Upper segment. Five sites are chosen between a stretch of 60+ km, and samples are collected during monsoon and post-monsoon seasons. Physico-chemical parameters (pH, Turbidity, Sulphate, Phosphate, Fluoride, Chloride, Total Hardness, Calcium, Magnesium, Dissolved Oxygen, BOD, COD, Alkalinity), water quality index and Pearson correlation coefficient were calculated for all chosen sites. Since the study was initiated during COVID, initial results show the impact of reduced industrial and urban activities in improving the overall water quality.

## Introduction

Water is the most vital resource for human existence and for developmental activities that take place around the world (Wan Mohtar 2019). Rivers have been the prime source of water since ancient times. Cities and industries have developed close to rivers. Along with industrial usage, rivers act as a major source for irrigation and daily needs (Meybeck 1976; Ridoutt and Pfister 2010; Somashekar et al. 2010). Even today, close to 50% of the world’s population is residing within 3 km of some surface freshwater source (Matti et al. 2011). While rivers have contributed to the overall development of the world, it has put a lot of pressure on the quality of water because of increased pollution levels. Over time, there has been deterioration in water quality due to natural and anthropogenic factors (Vadde et al. 2018; Nitasha and Sanjiv 2014). Deterioration of water quality has impacted the human as well as aquatic life (Aswal et al. 2016; Jindal and Sharma 2010; Kamboj et al. 2017; Sreebha and Padmalal 2011). Because of the importance that fresh water holds for humans and deterioration that has been happening since decades, it becomes important to assess the water quality on regular intervals and take appropriate measures to improve it.

Year 2020 holds and will continue to hold a lot of mind-space, as it has completely changed the way whole world operates. COVID-19, declared as an International Emergency of Public Health by World Health Organization (Kambalgere 2020; Sohrabi et al. 2020), has deeply impacted the economic development of twenty-first century. While the sudden lockdowns across countries have harmed the economic activities, overall environment has benefitted because of limited anthropogenic activities (Beine et al. 2020; Ray et al. 2020; Huang et al. 2020). Various studies done across nations showed significant ecological restoration (Ma et al. 2020; Yongjian et al. 2020; Sharma et al. 2020b; Gautam and Hens 2020; Bera et al. 2020).

Water quality of rivers in India has also significantly improved (quality of river Ganga improved by 40–50% during lockdown period) (CPCB 2020, Mani 2020). Studies done on water quality across many sites in India show that parameters like pH, DO, BOD, and TC have improved considerably (Dhar et al. 2020, Chakraborty et al. 2020; Mukherjee et al. 2020).

Water pollution of any area/site can be analysed by measuring various physical, chemical and biological parameters (Vasistha and Ganguly 2020a, b). It has been noticed that use of individual variables cannot completely describe water quality (Bharti and Katyal 2011). Water quality is therefore evaluated by a water quality index (WQI), a single number derived from many water quality parameters (Nives 1999; Pesce and Wunderlin 2000). There are various methods to calculate the water quality index: weighted arithmetic index method (Brown et al. 1970), National Sanitation Foundation Water Quality Index (NSFWQI) (Hoseinzadeh et al. 2014), overall index of pollution (OIP) (Sargaonkar and Deshpande 2003), etc. In our study, weighted arithmetic index method is used to calculate WQI. This is the most comprehensive way to measure water pollution where bulk of data is reduced to a single value to express information in simple way. The standards used are the ones recommended by Bureau of Indian Standards (BIS 2012).

## Study area

**Fig. 1:**
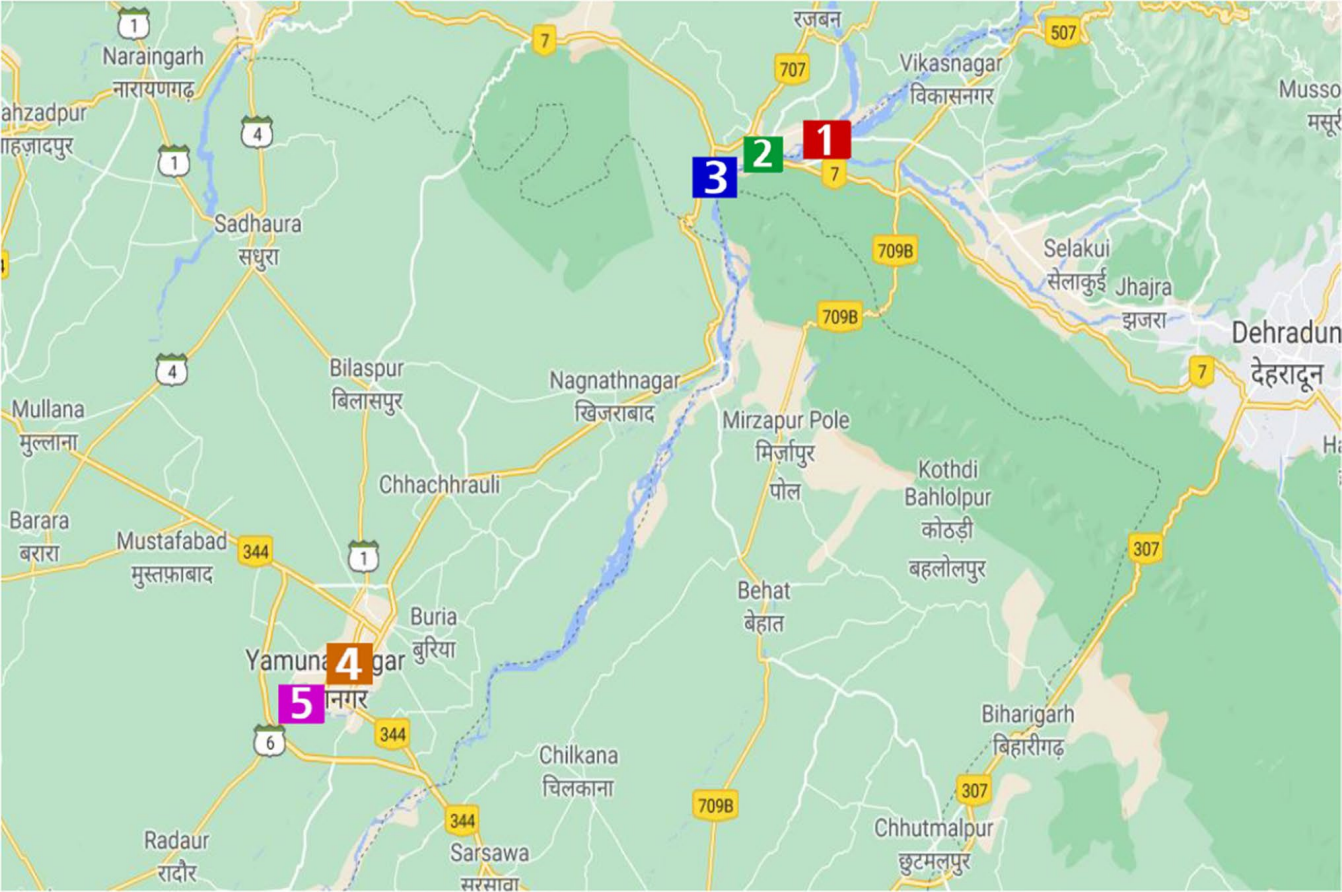
Map of study area

The study is done between a distance of 65+ km. Sampling sites have not only been selected from origin and end points but in between the stretch too. Study aims to show variation in various physico-chemical parameters in Himalayan and Upper Segments, where variation in water quality could happen due to influx of various pollutants. Variation in water quality index is analysed to show the impact of pollutants within the mentioned range, which can be of help to improve the situation at specific sites. Description of 5 sample sites in three states—Uttarakhand, Himachal Pradesh and Haryana—is given in Table 1 and Fig. 1.

**Table 1:** Description of sampling sites

Site No	Site location	State
1	Dhalipur, Chakrata Road, Vikasnagar	Uttarakhand
2	Swarg Dham, Nr Telephone Exchange, Paonta Sahib	H.P
3	Timli Range, Bhuppur, Paonta Sahib	H.P
4	Vishwakarma Mohalla, Yamuna Nagar	Haryana
5	Old Hamida, Yamuna Nagar	Haryana

## Methodology

Samples were collected from each site in September and December’20. September was the monsoon month with limited industrial activity and commercialization because of COVID. Another lot of samples were collected in December’20 (Post-Monsoon period). Most of the industrial and commercial activities had started (similar to the Pre-COVID times). Samples were collected from 50 cm depth at each site. Pre-cleaned and De-ionized bottles were used to collect the samples and were preserved in a proper way. Various physico-chemical parameters were considered to assess the pollution levels/water quality. Temperature and dissolved oxygen were calculated at sample collection points. Methods recommended by APHA (23rd edition) were used for analysis. pH was analysed by pH Meter (Systronics pH System 361). Electrical conductivity was measured using Conductivity Meter (Systronics Conductivity Meter 306). Total hardness was measured using EDTA Titrimetric method (IS 3025). TDS was calculated using 2540-C method, and TSS was calculated using 2540-D. Biological oxygen demand (BOD) was calculated by incubation of sample for 5 days (APHA 5210-B) and chemical oxygen demand (COD) by closed reflux method (522C and D). Spectrophotometer was used to detect fluoride (Spadns method) and nitrate. Titrimetric method was used to analyse Chloride (Arginometric method).

### Calculation of water quality index (WQI) using weighted arithmetic method

- *Step* 1: Collect data of various physico-chemical water quality parameters.
- *Step* 2: Calculate Proportionality constant “*K*” value using formula;
  $$K = \left[ {\frac{1}{{\frac{1}{{}}\sum n_{i} s_{i} }}} \right]$$
  where “*s*_i_” is standard permissible for n^th^ parameter.
- *Step* 3: Calculate quality rating for n^th^ parameter (q_n_) where there are n parameters. This is calculated using formula $q_{n} = 100\left\{ {\frac{{v_{n} - v_{{{\text{io}}}} }}{{s_{n} - v_{{{\text{io}}}} }}} \right\}$. where *v*_*n*_ = Estimated value of the *n*th parameter of the given sampling station. *v*_io_ = Ideal value of *n*th parameter in pure water. And *s*_*n*_ = Standard permissible value of the *n*th parameter.
- *Step* 4: Calculate unit weight for the *n*th parameters. *W*_*n*_ = (*k*/*s*_*n*_).
- *Step* 5: Calculate water quality index (WQI) using formula, $WQI = \frac{ \Sigma wn * q n }{{\Sigma wn}}.$
- Water quality status is judged on the basis of numerical value received from WQI depicted in Table 2.

**Table 2:** Various Index levels and their corresponding water quality

Water quality index level	Water quality status	Possible usage
0–25	Excellent	Drinking, irrigation, industrial
26–50	Good	Drinking, irrigation, industrial
51–75	Poor	Irrigation and industrial
76–100	Very poor	Irrigation
> 100	Unsuitable for drinking	Treatment required before use

### Results and discussions

Results of all the analysed physico-chemical parameters during the monsoon and post-monsoon seasons are shown in Table 3 (BIS 2012; Pipraiya et al. 2017).

**Table 3:** Results of analysed physico-chemical parameters in monsoon and post-monsoon period

Parameter	Standards	Monsoon					Post-monsoon				
	BIS-2012	Site 1	Site 2	Site 3	Site 4	Site 5	Site 1	Site 2	Site 3	Site 4	Site 5
pH	8.5	7.2	7.3	7.3	7.5	7.9	7.5	7.6	7.7	7.8	7.9
Turbidity	10	3	4.1	5.3	7	6.5	3.1	3.4	3.3	8.5	9
Conductivity	300	206.4	212.4	219.6	264.1	279.1	277	284.8	300.75	232.9	349.6
TDS	500	215	169	157	175	164	288	288	267	202	254
TSS	500	6	10	8	28	32	2	37	6	39	42
Nitrate	45	0.4	0.45	0.41	0.66	0.85	0.81	0.84	0.85	1.06	1.67
Fluoride	1.5	0.39	0.36	0.36	0.39	0.49	0.37	0.34	0.41	0.7	0.98
Sulphate	150	25.3	25.78	28.7	22.12	28.64	16.98	36.86	37.89	30.05	33.03
Phosphate	5	0.13	0.14	0.14	0.16	0.16	0.09	0.12	0.14	0.12	0.12
Chloride	250	6.2	9.94	7.1	14.2	24.14	11.36	12.78	12.21	11.36	22.72
Total hardness	200	123	138	126	134	134	138	200	182	124	168
Calcium hardness	75	96	80	106	100	84	90	130	112	78	104
Magnesium hardness	30	27	58	20	34.01	50	48	70	70	46	64
DO	5	8.4	7.6	8	7.3	7.1	8.5	6.7	8.2	7.7	5.2
BOD	5	0.7	0.8	0.7	0.9	1	0.2	0.9	0.5	1.1	2.8
COD	5	2.4	2.4	1.6	4	4.8	1.6	2.4	1.6	4.8	5.6
Total alkalinity	120	360	245	300	245	230	135	170	150	105	165
Bicarbonate	120	360	245	300	245	230	135	170	150	105	165

Conductivity shows higher values than the standards in Site 5 during post-monsoon season. Calcium hardness, magnesium hardness, DO and total alkalinity are more than the permissible limits set by BIS (Bureau of Indian Standards) in both monsoon and post-monsoon season.

The water quality index for various sites of the river Yamuna was calculated from thirteen parameters, namely pH, turbidity, conductivity TDS, TSS, nitrate, chloride, total hardness, calcium hardness, magnesium hardness, total alkalinity, DO and sulphate. Five sampling stations were chosen to assess the aptness of river water for general purposes.

The descriptive statistics of these physico-chemical water quality parameters for each site were considered (Singh et al. 2018; Vasistha and Ganguly 2020a, b) and calculated by adopting weighted arithmetic method and given in Tables 4 and 5.

**Table 4:** Standard values and standard weights of various parameters

Parameters	Standard value (*S*_*n*_)	Standard weight (*W*_*n*_)
pH	8.5	0.2272
Turbidity	10	0.1931
Conductivity	300	0.0064
TDS	500	0.0039
TSS	500	0.0039
Nitrate (as Nitrogen)	45	0.0429
Chloride	250	0.0077
Total hardness	200	0.0097
Calcium hardness	75	0.0257
Magnesium hardness	30	0.0644
Total alkalinity	120	0.0161
DO	5	0.3862
Sulphate	150	0.0129

**Table 5:** Water Quality Index (WQI) of sites during monsoon and post-monsoon season

Parameters	Monsoon season					Post-monsoon season				
	Site 1	Site 2	Site 3	Site 4	Site 5	Site 1	Site 2	Site 3	Site 4	Site 5
pH	3.03	4.54	4.54	7.57	13.63	7.57	9.09	10.60	12.12	13.63
Turbidity	5.79	7.92	10.23	13.52	12.55	5.99	6.57	6.37	16.41	17.38
Conductivity	0.44	0.46	0.47	0.57	0.60	0.59	0.61	0.65	0.50	0.75
TDS	0.17	0.13	0.12	0.14	0.13	0.22	0.22	0.21	0.16	0.20
TSS	0.00	0.01	0.01	0.02	0.02	0.00	0.03	0.00	0.03	0.03
Nitrate	0.04	0.04	0.04	0.06	0.08	0.08	0.08	0.08	0.10	0.16
Chloride	0.02	0.03	0.02	0.04	0.07	0.04	0.04	0.04	0.04	0.07
Total hardness	0.59	0.67	0.61	0.65	0.65	0.67	0.97	0.88	0.60	0.81
Calcium hardness	3.30	2.75	3.64	3.43	2.88	3.09	4.46	3.84	2.68	3.57
Magnesium hardness	5.79	12.44	4.29	7.30	10.73	10.30	15.02	15.02	9.87	13.73
Total alkalinity	4.83	3.29	4.02	3.29	3.08	1.81	2.28	2.01	1.41	2.21
DO	24.94	28.16	26.55	29.37	30.17	24.54	31.78	25.75	27.76	37.82
Sulphate	0.22	0.22	0.25	0.19	0.25	0.15	0.32	0.33	0.26	0.28
WQI(Σ*W*_*n*_*Q*_*n*_)	49.16	60.65	54.80	66.14	74.85	55.04	71.46	65.77	71.92	90.64

The above values depict that water quality index of 5 sites under analysis show deterioration during the post-monsoon season. This is against the regular trend where monsoon season usually witnesses higher WQI than the post-monsoon season. The primary reason attributed to this variation is the impact of COVID on industrial, commercial and tourist activities. Since the industrial activities were less because of precautionary/administrative measures, the pollution through discharge of effluents was also minimal. To add on, there was less movement of people to work and leisure.

**Fig. 2:**
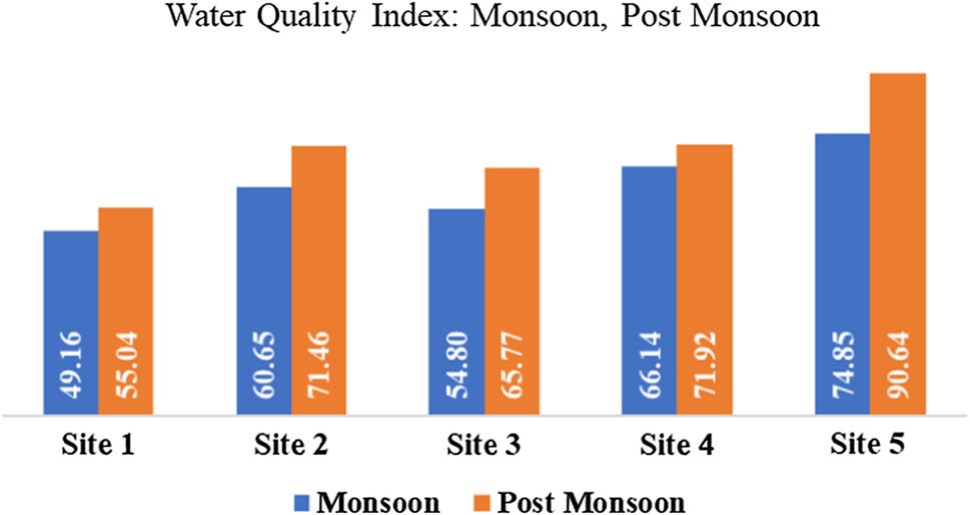
Variation of water quality index for sites in monsoon and post-monsoon season

Water quality index of all sites in post-monsoon season is higher than monsoon season. While most of the sites show poor Water Quality Index, there is a major variation at Site 5 (WQI changed from poor to very poor as shown in Table 6 and Fig. 2.

**Table 6:** Summary of water quality in monsoon and post-monsoon seasons

Season/Site	Site 1	Site 2	Site 3	Site 4	Site 5
Monsoon	49.16 (Good)	60.65 (Poor)	54.80 (Poor)	66.14 (Poor)	74.85 (Poor)
Post-monsoon	55.04 (Poor)	71.46 (Poor)	65.77 (Poor)	71.92 (Poor)	90.64 (Very poor)

### Correlation between various parameters

Correlation between various variables is depicted through Pearson correlation coefficient. It is a statistical tool that measures the relationship between various variables (physico-chemical parameters in our study). It provides information on magnitude of correlation and the direction of its relationship. (Tables 7 and 8).

**Table 7:** Correlation among various parameters during monsoon season

Parameters	pH	Turbidity	Conductivity	TDS	TSS	Nitrate	Sulphate	Chloride	Total hardness	Calcium hardness	Magnesium hardness	Dissolved Oxygen	Total alkalinity
pH	1												
Turbidity	0.74	1											
Conductivity	0.93	0.90	1										
TDS	− 0.44	− 0.64	− 0.41	1									
TSS	0.91	0.87	0.99	− 0.36	1								
Nitrate	0.98	0.78	0.97	− 0.35	0.97	1							
Sulphate	0.26	− 0.08	− 0.04	− 0.42	− 0.14	0.05	1						
Chloride	0.99	0.71	0.92	− 0.40	0.92	0.98	0.18	1					
Total hardness	0.46	0.45	0.45	− 0.54	0.53	0.50	− 0.19	0.55	1				
Calcium hardness	− 0.36	0.14	− 0.13	0.06	− 0.22	− 0.34	− 0.13	− 0.47	− 0.68	1			
Magnesium hardness	0.43	0.08	0.27	− 0.25	0.36	0.43	0.01	0.54	0.86	− 0.96	1		
Dissolved Oxygen	− 0.84	− 0.83	− 0.87	0.62	− 0.90	− 0.87	0.06	− 0.87	− 0.82	0.43	− 0.62	1	
Total alkalinity	− 0.70	− 0.74	− 0.70	0.74	− 0.74	− 0.71	0.02	− 0.74	− 0.92	0.49	− 0.70	0.96	1

**Table 8:** Correlation among various parameters during post-monsoon season

Parameters	pH	Turbidity	Conductivity	TDS	TSS	Nitrate	Sulphate	Chloride	Total hardness	Calcium hardness	Magnesium hardness	Dissolved Oxygen	Total alkalinity
pH	1												
Turbidity	0.89	1											
Conductivity	0.35	0.11	1										
TDS	− 0.69	− 0.79	0.42	1									
TSS	0.67	0.74	0.08	− 0.49	1								
Nitrate	0.85	0.84	0.63	− 0.36	0.62	1							
Sulphate	0.48	0.10	0.28	− 0.12	0.43	0.16	1						
Chloride	0.69	0.61	0.84	− 0.03	0.50	0.94	0.23	1					
Total hardness	− 0.08	− 0.42	0.54	0.61	0.08	− 0.06	0.71	0.22	1				
Calcium hardness	− 0.19	− 0.48	0.48	0.67	0.08	− 0.14	0.64	0.15	0.99	1			
Magnesium hardness	0.11	− 0.30	0.63	0.48	0.09	0.07	0.80	0.32	0.97	0.92	1		
Dissolved Oxygen	− 0.66	− 0.61	− 0.67	0.07	− 0.79	− 0.84	− 0.42	− 0.90	− 0.37	− 0.33	− 0.40	1	
Total alkalinity	− 0.03	− 0.28	0.80	0.71	0.11	0.21	0.45	0.52	0.89	0.89	0.86	− 0.57	1

The above correlation matrix indicates that highly positive correlation values for conductivity with pH and turbidity; TSS with pH, turbidity and conductivity; nitrate with pH, turbidity, conductivity and TSS; Chloride with pH, turbidity, conductivity, TSS and nitrate; magnesium hardness with total hardness and total alkalinity with DO in monsoon season.

In post-monsoon season, highly positive correlation exists between turbidity with pH; nitrate with pH and turbidity; chloride with conductivity and nitrate; calcium hardness with total hardness; magnesium hardness with sulphate, total hardness and calcium hardness; total alkalinity with total hardness, and calcium hardness.

## Conclusion

COVID-19 has had a significant impact on the overall environment. This research signifies its impact on the Water Quality Index of river Yamuna during the monsoon season and compares it with the post-monsoon season. Pollution arising from various sources significantly impacts the water quality. In this study, the primary focus was on thirteen water quality parameters (pH, Turbidity, Sulphate, Phosphate, Fluoride, Chloride, Total Hardness, Calcium, Magnesium, Dissolved Oxygen, BOD, COD and Alkalinity) to illustrate impact of rising pollution levels of river water quality from five sampling stations. Conductivity shows higher values than the standards in Site 5 during post-monsoon season. Total hardness is on specified limit in Site 2 during post-monsoon season. Calcium hardness shows higher than standard values for all sites during monsoon and post-monsoon season. Magnesium hardness is more than limits at Site 2, 4 and 5 during monsoon season and across all sites in post-monsoon season. D.O and alkalinity show higher values than limits across all sites during both seasons. Emphasis was also given on comparison of WQI during monsoon and post-monsoon season. Water quality index (WQI) during monsoon season usually shows deterioration. However, because of minimal industrial activities and low tourism during COVID, there is a significant improvement in overall parameters at all sites. As normal activities start post opening up by the administration, the water quality index (WQI) shows deterioration during the post-monsoon season, when compared with monsoon season. Correlation coefficient depicts the relation of various parameters with each other.

## Data Availability

All data and material related to this paper are readily available.
